# Characterization of arrhythmia‐induced cardiomyopathy using magnetic resonance imaging in patients with persistent atrial fibrillation and left ventricular systolic dysfunction – insights from DECAAF II

**DOI:** 10.1002/ejhf.3684

**Published:** 2025-05-12

**Authors:** Ala Assaf, Han Feng, Mayana Bsoul, Ghassan Bidaoui, Hadi Younes, Christian Massad, Mario Mekhael, Charbel Noujaim, Omar Kreidieh, Swati Rao, Amitabh Pandey, Philipp Sommer, Christian Mahnkopf, Nassir Marrouche, Christian Sohns

**Affiliations:** ^1^ Tulane Research Innovation for Arrhythmia Discovery (TRIAD) Tulane University School of Medicine New Orleans LA USA; ^2^ Cleveland Clinics Foundation Cleveland OH USA; ^3^ Clinic for Electrophysiology, Herz‐ und Diabeteszentrum NRW Ruhr‐Universität Bochum, Med. Fakultät OWL (Universität Bielefeld) Bad Oeynhausen Germany; ^4^ Department of Cardiology, Klinikum Coburg Coburg Germany

**Keywords:** Atrial fibrillation, Atrial fibrosis, Atrial myopathy, Ejection fraction, Heart failure, Ventricular dysfunction

## Abstract

**Aims:**

Atrial fibrillation (AF) ablation in heart failure reduces mortality and hospitalizations and improves ejection fraction. Arrhythmia‐induced cardiomyopathy (AIC) is diagnosed after complete recovery of left ventricular systolic function after ablation. We aimed to identify the prevalence and pre‐ablation predictors of AIC among patients with AF and left ventricular systolic dysfunction (LVSD).

**Methods and results:**

We utilized the DECAAF II database, where 815 patients with persistent AF underwent late gadolinium enhancement cardiac magnetic‐resonance imaging (LGE‐CMR) before and 3 months after AF ablation. We only included patients with available left ventricular ejection fraction (LVEF) and LVSD. AF burden was continuously monitored. AIC was defined as LVSD and coexisting AF in patients with ejection fraction improvement to ≥50% following ablation. We identified 119 patients with LVSD and AF with a mean LVEF of 39.1 ± 7.8% and mean baseline fibrosis of 20.0 ± 7.3%. Mean AF burden post‐ablation was 16.8 ± 20.2%, and mean LVEF recovery was 13.9 percentage points. Seventy‐two patients (60.5%) fulfilled the criteria for AIC, and 47 (39.5%) did not. AIC patients had a mean baseline LVEF of 39.1 ± 7.9% (vs. 39.2 ± 7.9% in non‐AIC patients; *p* = 0.9), a significantly lower percentage of fibrosis in the left atrial septal wall (12.2 ± 10.0% vs. 20.7 ± 11.4% in non‐AIC patients, *p* < 0.001). Additionally, LVEF improvement was correlated with lower AF burden post‐ablation (*r* = −0.23, *p* = 0.02).

**Conclusions:**

In this post‐hoc analysis of the DECAAF II trial, we found that the majority of patients with LVSD and persistent AF have AIC rather than primary cardiomyopathy. We identified LGE‐CMR as a differentiator between AIC and other cardiomyopathies.

## Introduction

Catheter ablation (CA) for atrial fibrillation (AF) in patients with heart failure and reduced ejection fraction (HFrEF) has recently received a class I indication as a first‐line therapy.^1^ This recommendation stems from evidence presented in clinical trials such as CASTLE‐AF,^2^ and CASTLE‐HTx,^3^ along with post‐hoc analyses of the CABANA and EAST‐AFNET trials.^4^, ^5^ These investigations have consistently shown that AF ablation significantly reduces mortality and hospitalizations while improving ejection fraction in HF patients with AF.

Atrial fibrillation and left ventricular systolic dysfunction (LVSD) frequently coexist, with AF serving both as a consequence and a precipitant factor of LVSD and HF.^6^ Despite these interactions, the exact mechanisms driving favourable outcomes of AF ablation in HF patients are not fully understood. A prominent hypothesis is that LVSD, and subsequent HF in these patients may be secondary to AF, leading to a condition known as arrhythmia‐induced cardiomyopathy (AIC), which can be completely resolved with AF ablation.^7^, ^8^ However, the prevalence of HF being secondary to AF in this population remains uncertain.^8^ Furthermore, the current diagnosis of AIC is typically made retrospectively, only after observing the recovery of systolic function following ablation.^8^ This limitation leaves clinicians without a reliable method to preoperatively identify which patients have AIC.

Given these challenges, we aimed to address two critical gaps in the current understanding of AIC in LVSD patients with AF, utilizing the Effect of MRI‐Guided Fibrosis Ablation versus Conventional Catheter Ablation on Atrial Arrhythmia Recurrence in Patients with Persistent Atrial Fibrillation (DECAAF II) trial database.^9^ First, we sought to determine the prevalence of AIC among patients with AF and LVSD. Second, we aimed to identify pre‐ablation predictors of AIC. Specifically, we aimed to investigate the use of atrial late gadolinium enhancement cardiac magnetic resonance (LGE‐CMR) in identifying a fibrosis fingerprint of AIC.

## Methods

### Patient population

We used the DECAAF II trial database for this analysis.^9^ Details of the study design, patients, outcome definitions, and results have been published.^9^ Briefly, 815 patients with persistent AF undergoing first‐time CA for AF were randomized to receive pulmonary vein isolation (PVI) or magnetic resonance imaging (MRI) fibrosis‐guided substrate modification in addition to PVI (PVI + SM). This study was approved by the Tulane University Biomedical Institutional Review Board and complies with the Declaration of Helsinki. For this analysis, we only included patients with LVSD with available left ventricular ejection fraction (LVEF) data prior to and 3 months after ablation. LVSD was defined as patients with pre‐ablation LVEF ≤50%, as detailed in the DECAAF II protocol.

We defined AIC as LVSD and AF patients who experience ejection fraction improvement to ≥50% after ablation with at least 10% absolute improvement in ejection fraction, or an absolute improvement ≥15%.^10^ All other patients with AF and LVSD were defined as non‐AIC. Patients with both ischaemic and non‐ischaemic cardiomyopathy were included.

### Left ventricular ejection fraction acquisition

Left ventricular ejection fraction was obtained using a regular cardiac transthoracic echocardiography or the cine sequence of the LGE‐MRI cine MRI at baseline and at 3 months post‐ablation, according to the guidelines.^11^, ^12^ HF symptoms and status, guideline‐directed medical therapy (GDMT) and antiarrhythmic drugs (AADs) were collected from electronic medical records.

### Rhythm monitoring and atrial fibrillation burden

Patients were followed up for 12–18 months using a mobile, handheld single‐lead electrocardiogram (ECG Check Device, Cardiac Designs Inc.) and were asked to submit at least one strip per day or when feeling symptoms suggestive of arrhythmia recurrence. AF recurrences were defined as any atrial tachyarrhythmia lasting for at least 30 s, as documented by the mobile ECG, standard 12‐lead ECG, ambulatory Holter monitoring, or repeat ablation after the 90‐day blanking period. AF burden was calculated as the number of days with documented AF divided by the total days monitored, as previously reported.^13^

### Image acquisition and processing

All patients underwent cardiac LGE‐MRI using previously described methods.^9^, ^14^, ^15^, ^16^ The left atrial (LA) wall was manually segmented, and regions of fibrosis were delineated using an intensity threshold set by expert inspection of each image. Regions exhibiting enhancement intensity two to three standard deviations above the mean intensity of normal tissues were considered fibrotic, using the Corview image analysis software (MARREK, Inc., Salt Lake City, UT, USA).^16^ The amount of fibrosis in the left atrium was stratified into the four Utah stages.^17^ Baseline fibrosis was defined as the total amount of fibrosis in the LA wall prior to ablation divided by the surface area of the LA wall. The LA wall was divided into seven regions (anterior, posterior, lateral, septal, left atrial appendage, left pulmonary vein [PV] ostia, right PV ostium) according to previously described methods.^16^, ^18^

### Statistical analysis

All categorical variables were reported as frequencies and percentages and compared using the *χ* ^2^ test. Continuous variables were reported as means and standard deviations and compared using Mann–Whitney U tests and Student's *t*‐tests based on whether the normality assumptions were rejected by Shapiro–Wilk tests or not, respectively. We addressed missing data by including only complete cases in the analysis without applying any imputation techniques, considering the rare occurrence of missing data (11 subjects missing post‐MRI information due to low quality) within the study population. The correlation between AF burden and AIC status was assessed through a multivariable logistic model adjusting for diabetes mellitus, stroke, hyperlipidaemia, follow‐up ablation scar, and baseline fibrosis. We further found a cutoff of 3.8% for AF burden as an optimal predictor of AIC status through grid searching based on the largest AUC provided by the multivariable model. Univariable logistic models were applied to all relevant variables for screening purposes. All the variables with *p* < 0.2 were further developed into the multivariable logistic model. A two‐sided *p*‐value of 0.05 was considered statistically significant. Analyses were done using Python (version 3.7) and R (version 4.3.0).

## Results

### Baseline characteristics

We identified 119 patients with LVSD and available ejection fraction data prior to and after ablation. Patients with HFpEF were excluded. The average age of these patients was 61.8 ± 9.9 years, and 85.7% were male. Their average body mass index was 31.7 ± 6.6 kg/m^2^. Hypertension was present in 59.7% of patients, diabetes mellitus was found in 6.7%, and coronary artery disease was prevalent in 16.0% of patients. 56.3% of patients were randomly assigned to receive PVI alone, whereas 43.7% were assigned to receive PVI + SM. Prior to ablation, patients had baseline fibrosis from LGE‐CMR of 20.0 ± 7.3%, LVEF of 39.1 ± 7.9%, LA volume of 145.3 ± 40.6 mm^3^, and heart rate (HR) of 115.8 ± 21.9 bpm. Further patient information is shown in *Table* *1*.

**Table 1:** Summary characteristics

	Total (*n* = 119)	Non‐AIC (*n* = 47)	AIC (*n* = 72)	*p*‐value
Treatment randomization, *n* (%)				0.56
Conventional PVI	67 (56.3)	28 (59.6)	39 (54.2)	
PVI + fibrosis‐guided ablation	52 (43.7)	19 (40.4)	33 (45.8)	
Age, years, mean (SD)	61.79 (9.90)	61.55 (10.76)	61.94 (9.37)	0.87
Sex, *n* (%)				0.36
Female	17 (14.3)	5 (10.6)	12 (16.7)	
Male	102 (85.7)	42 (89.4)	60 (83.3)	
Body mass index, kg/m^2^, mean (SD)	31.70 (6.58)	31.23 (7.18)	32.01 (6.18)	0.93
Hypertension, *n* (%)	71 (59.7)	29 (61.7)	42 (58.3)	0.71
Diabetes mellitus, *n* (%)	8 (6.7)	5 (10.6)	3 (4.2)	0.17
Stroke, *n* (%)	8 (6.7)	5 (10.6)	3 (4.2)	0.17
History of tobacco use, *n* (%)	46 (38.7)	16 (34.0)	30 (41.7)	0.40
Coronary artery disease, *n* (%)	19 (16.0)	7 (14.9)	12 (16.7)	0.80
History of vascular disease, *n* (%)	13 (10.9)	6 (12.8)	7 (9.7)	0.60
Hyperlipidaemia, *n* (%)	50 (42.0)	15 (31.9)	35 (48.6)	0.071
Long‐standing persistent AF (out of patients with available persistent vs. long‐standing persistent AF), *n* (%)	39 (39.4)	12 (31.6)	27 (44.1)	0.878
Antiarrhythmic medication, *n* (%)	54 (45.4)	22 (46.8)	32 (44.4)	0.80
Prior antiarrhythmic treatment, *n* (%)	72 (60.5)	25 (53.2)	47 (65.3)	0.19
Baseline fibrosis, %, mean (SD)	20.00 (7.33)	21.36 (7.05)	19.12 (7.42)	0.067
AF recurrence, *n* (%)	53 (44.5)	22 (46.8)	31 (43.1%)	0.69
LA volume pre‐ablation, mm^3^, mean (SD)	145.29 (40.56)	145.30 (45.83)	145.28 (37.05)	0.75
BNP, pg/ml, mean (SD)	443.55 (483.92)	492.48 (516.97)	397.50 (461.69)	0.58
LA volume reduction, mm^3^, mean (SD)	36.19 (28.72)	31.26 (28.17)	39.21 (28.85)	0.23
Follow‐up ablation scar, %, mean (SD)	8.06 (4.53)	7.38 (4.82)	8.48 (4.33)	0.095
Covered fibrosis, %, mean (SD)	0.20 (0.10)	0.20 (0.11)	0.20 (0.10)	0.56
AF burden post‐blanking period, %, mean (SD)	0.17 (0.20)	0.18 (0.23)	0.16 (0.19)	0.65
				0.91
Left ventricular ejection fraction pre‐ablation, %, mean (SD)	39.12 (7.85)	39.21 (7.92)	39.06 (7.86)	
Regional distribution of fibrosis, mean (SD)				
Posterior wall	21.75 (10.94)	23.15 (10.94)	20.83 (10.91)	0.26
Anterior wall	16.29 (10.08)	17.26 (9.22)	15.66 (10.62)	0.23
Lateral wall	22.91 (12.86)	24.24 (14.10)	22.04 (12.01)	0.59
Septal wall	15.55 (11.31)	20.69 (11.42)	12.20 (9.97)	<0.001
Right pulmonary vein ostia	14.64 (10.21)	15.31 (9.59)	14.20 (10.64)	0.41
Left pulmonary vein ostia	30.30 (14.36)	29.09 (13.07)	31.09 (15.18)	0.48
LA appendage	19.35 (10.74)	21.25 (10.61)	18.11 (10.73)	0.10

AF, atrial fibrillation; AIC, arrhythmia‐induced cardiomyopathy; BNP, B‐type natriuretic peptide; LA, left atrial; LV, left ventricular; PVI, pulmonary vein isolation; SD, standard deviation.

### Arrhythmia‐induced cardiomyopathy

**Figure 1:**
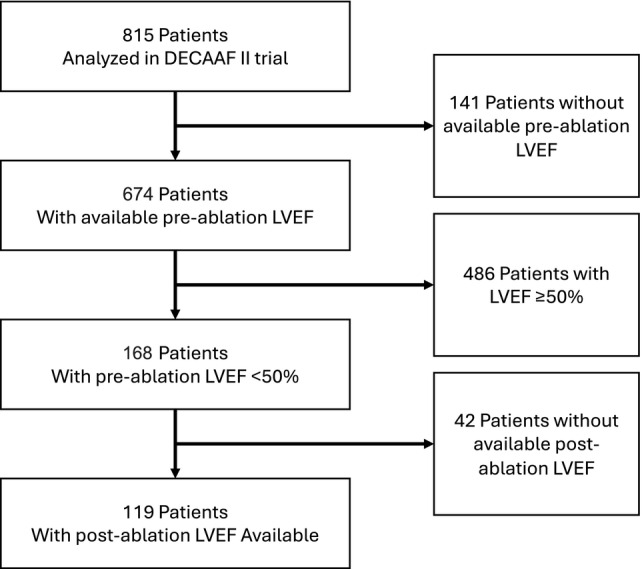
CONSORT flowchart for patient inclusion. LVEF, left ventricular ejection fraction.

**Figure 2:**
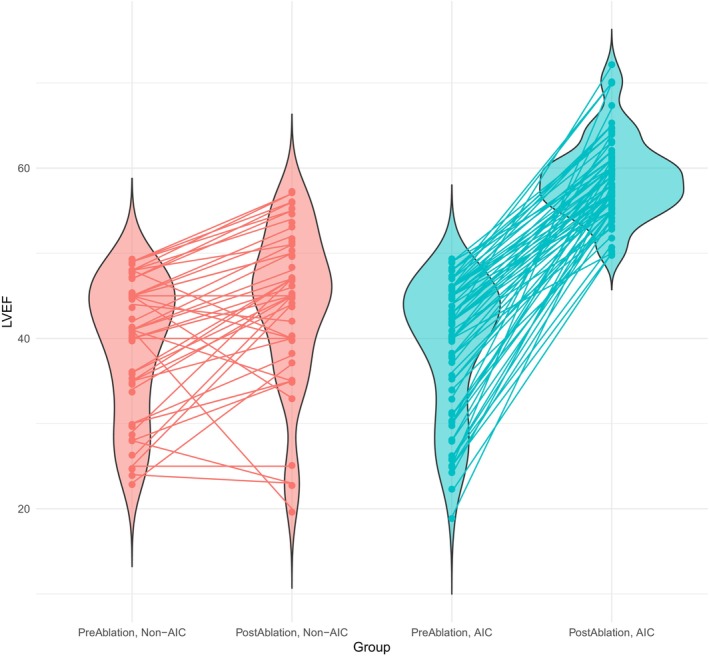
Box plot of pre‐ and post‐ablation left ventricular ejection fraction (LVEF) among different patient groups. AIC, arrhythmia‐induced cardiomyopathy.

**Figure 3:**
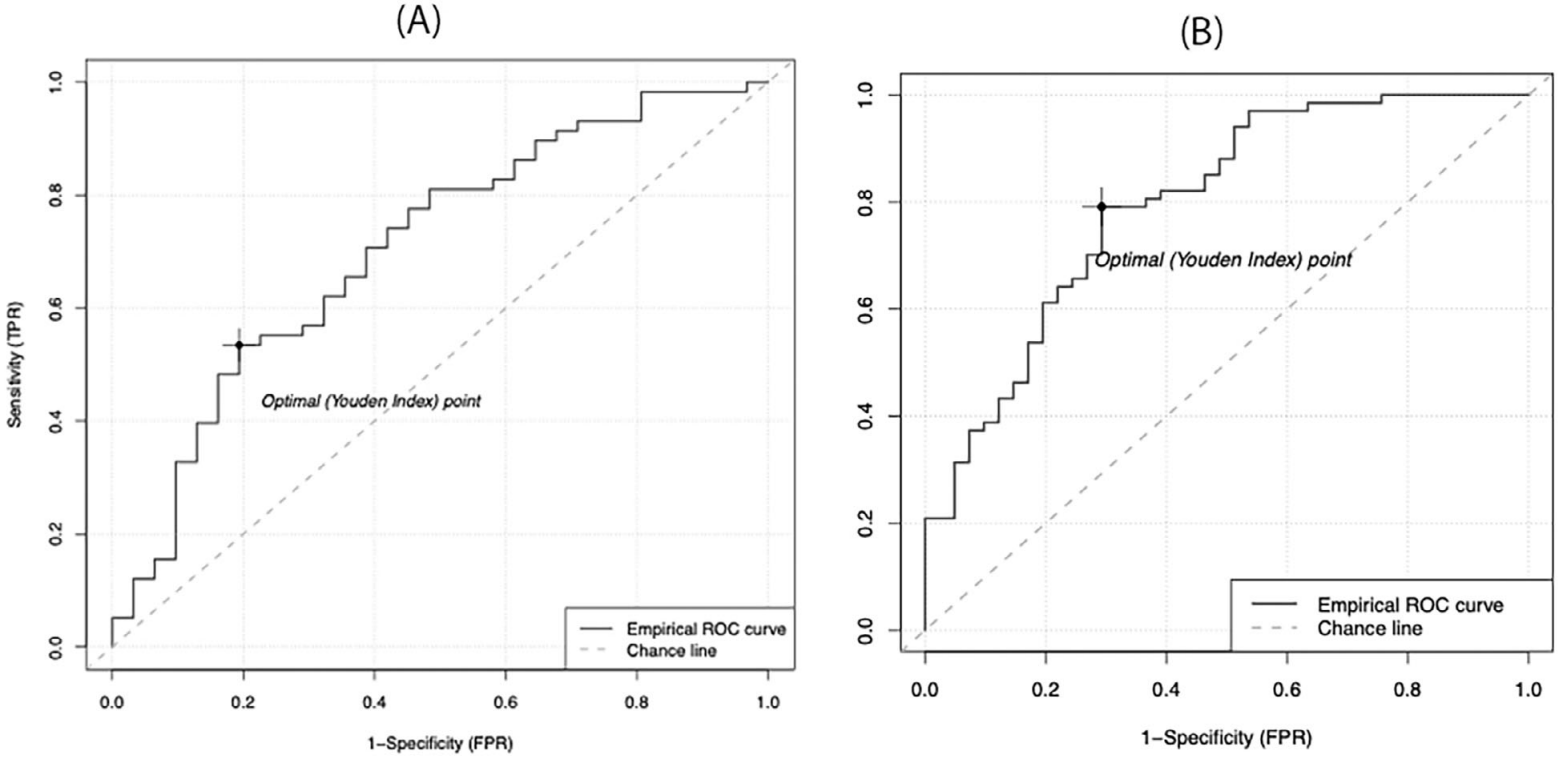
Receiver operating characteristic (ROC) curves demonstrating predictors of arrhythmia‐induced cardiomyopathy (AIC) status. (*A*) Atrial fibrillation (AF) burden predicting AIC status. (*B*) Septal reginal fibrosis predicting AIC status. FPR, false positive rate; TPR, true positive rate.

**Figure 4:**
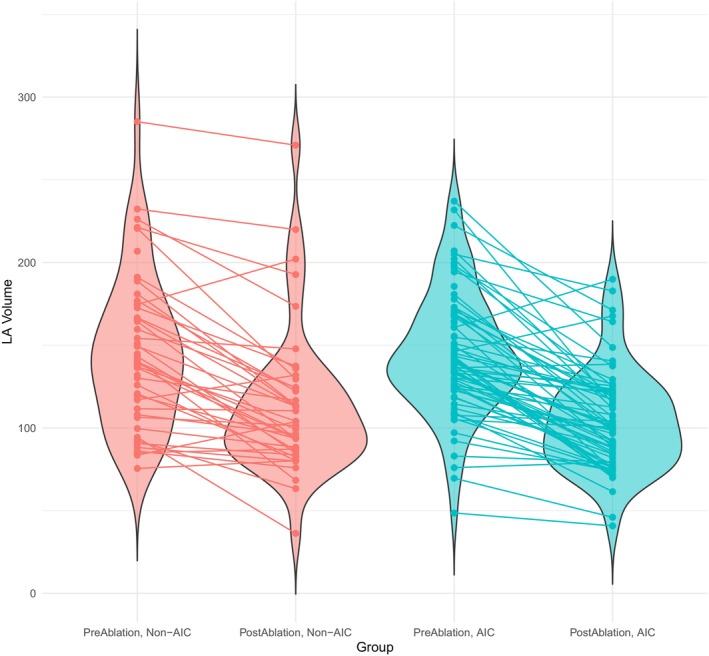
Left atrial (LA) volume reduction in both treatment groups. AIC, arrhythmia‐induced cardiomyopathy.

Seventy‐two (60.5%) of the 119 patients fulfilled the criteria for AIC, while 47 (39.5%) did not (*Graphical Abstract* and *Figure* *1*). At baseline, both groups had an average LVEF of 39% (*p* = 0.9). After ablation, patients fulfilling the AIC criteria improved in left ventricular function to a mean LVEF of 58.9 ± 4.7%, while patients not fulfilling the criteria had a mean LVEF of 44.0 ± 9.1% at 3 months after ablation (*p* < 0.001). In the AIC group, we observed an LVEF improvement by an average of 19.9 ± 7.6% compared to 4.8 ± 7.5% in non‐AIC patients (*p* < 0.001), with an average of 57.8% relative LVEF improvement (ejection fraction improvement divided by baseline ejection fraction) in AIC patients, and 14.3% in non‐AIC patients (*Figure* *2*). Substantial reductions in LA volume were observed in both groups following ablation (mean reduction = 36.2 ± 28.7 ml, *p* < 0.001) (*Figure* *3*). However, there was no significant difference in the extent of LA volume reduction between the two groups (31.3 ± 28.2 vs. 39.2 ± 28.9 ml, *p* = 0.23) (*Figure* *4*). Similarly, there was no difference between the two groups in GDMT or AAD use before or after ablation (online supplementary *Tables* *S1*–*S4*).

### Heart rate and rhythm

**Figure 5:**
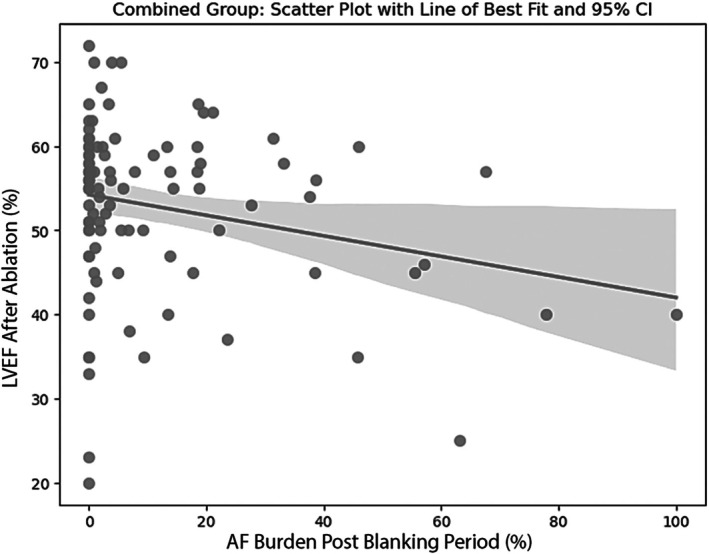
Correlation of atrial fibrillation (AF) with left ventricular ejection fraction (LVEF) post‐ablation. AF burden is defined as the amount of time spent in AF divided by the total time monitored after the 90‐day blanking period. Higher AF burden is associated with lower ejection fraction after ablation. CI, confidence interval.

Lower AF burden at 12 months post‐ablation was correlated with higher LVEF at 3 months post‐ablation (*r* = −0.23, *p* = 0.02) (*Figure* *5*). Specifically, we found that AF burden <3.8% was strongly predictive of AIC status (area under the curve [AUC] 0.706, *p* = 0.024) (*Figure* *3A*). However, there was no difference in AF recurrence (43.1% vs. 46.8%, *p* = 0.69) between the two groups.

There was a significant drop in HR post‐ablation for all patients (regardless of AIC status) (pre‐ablation: 113.9 ± 21.9 bpm, vs. post‐ablation: 90.1 ± 14.6 bpm, *p* < 0.05), but there was no difference in the magnitude of HR change between both groups (AIC −24.26 ± 28.30 bpm, non‐AIC = −22.77 ± 17.38 bpm, *p* = 0.8648). Similarly, there was no difference in pre‐ablation HR between the AIC and non‐AIC groups (114.26 ± 23.17 bpm vs. 119.35 ± 17.09 bpm, *p* = 0.485).

### Predictors of arrhythmia‐induced cardiomyopathy

At baseline, there was no significant difference between the two groups in age, sex, body mass index, or previous medical history. There was a trend towards less baseline fibrosis in the AIC group that did not reach statistical significance (*p* = 0.067).

**Figure 6:**
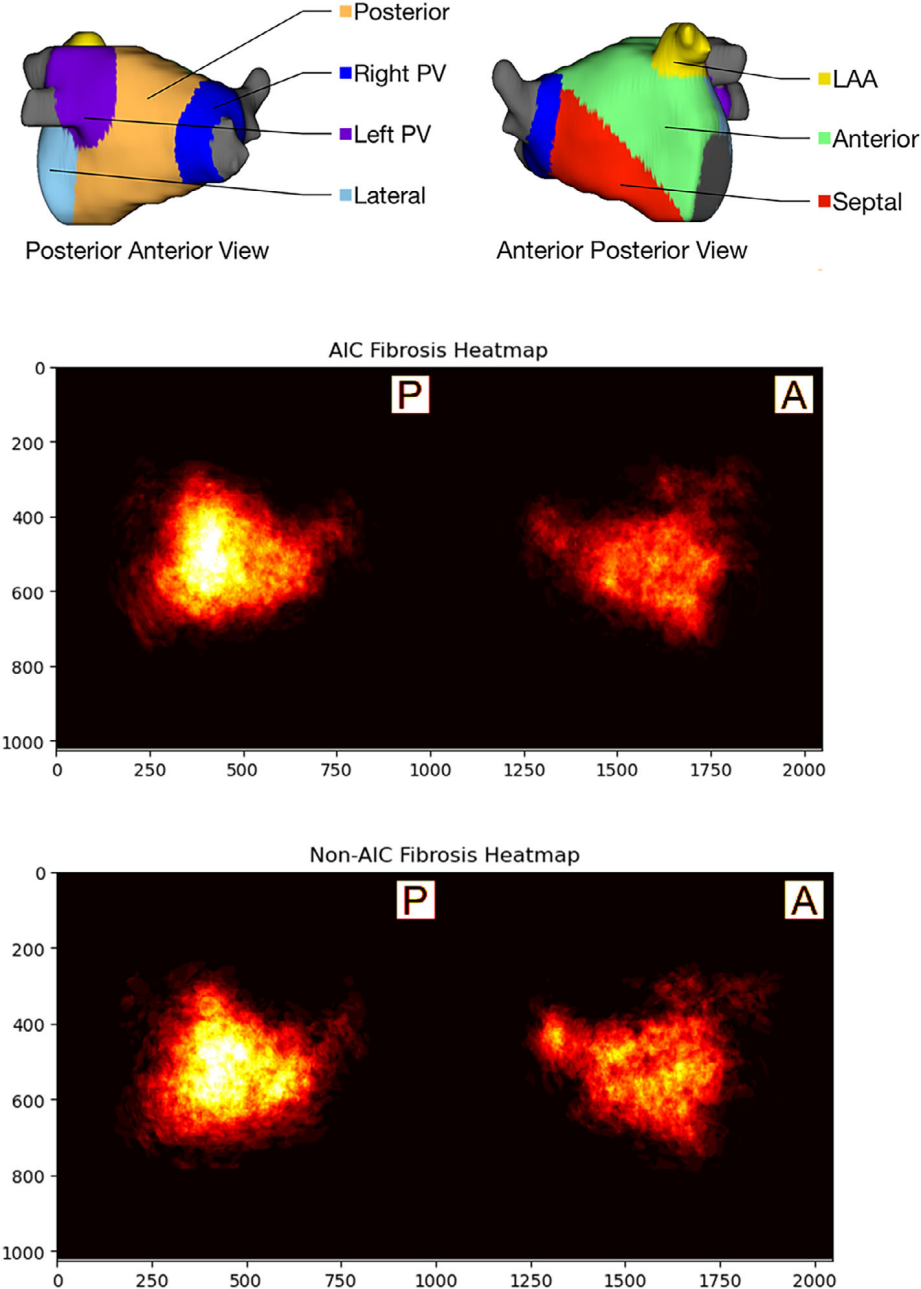
Heatmap distribution of fibrosis among the arrhythmia‐induced cardiomyopathy (AIC) and non‐AIC groups. Patients in the non‐AIC group have more fibrosis in the septal area of the left atrium. LAA, left atrial appendage; PV, pulmonary vein.

We found significant pre‐ablation differences between the AIC and non‐AIC groups in the regional distribution of fibrosis. Specifically, patients fulfilling the criteria for AIC had substantially lower amounts of fibrosis in the septal wall (AIC group 12.2 ± 10.0% vs. non‐AIC group 20.7 ± 11.4%; *p* < 0.001) (*Figure* *6*). There was a trend towards more hyperlipidaemia among AIC patients that barely missed significance (odds ratio 2.0, 95% confidence interval 0.9–4.3; *p* = 0.07). There was no significant difference between the two groups in the distribution of fibrosis in other segments of the LA wall. These findings remained significant in the multivariable logistic model (*Table* *2*). Logistic regression shows that septal regional fibrosis has an odds ratio of 0.92 for predicting AIC. In other words, each 1% increase in septal regional fibrosis reduces the likelihood of AIC by 8%. We identified a septal regional fibrosis cutoff of 15.8% through grid searching, which achieves an AUC of 0.80 in predicting AIC status (*p* < 0.001) (*Figure* *3B*).

**Table 2:** Multivariable model in predicting arrhythmia‐induced cardiomyopathy

	Estimate	Std. Error	*p*‐value	Odds ratio	95% CI
(Intercept)	0.769	0.658	0.243		
Diabetes mellitus	−0.907	0.867	0.296	0.404	(0.074– 2.210)
Stroke	−0.374	0.901	0.678	0.688	(0.118–4.022)
Hyperlipidaemia	0.829	0.442	0.061	2.290	(0.962–5.450)
Septal wall regional fibrosis	−0.087	0.026	0.001	0.917	(0.871–0.965)
Left atrial appendage regional fibrosis	−0.009	0.026	0.724	0.991	(0.942–1.043)
Baseline fibrosis	0.048	0.043	0.265	1.050	(0.964–1.143)

CI, confidence interval.

## Discussion

In this DECAAF II post‐hoc analysis, we present three major findings. Firstly, we identified that the majority of patients with concomitant persistent AF and LVSD have AIC. Secondly, we identified that less fibrosis of the interatrial septum as a predictor of AIC. Thirdly, we observed that the reduction in AF burden following ablation has a substantial impact on LVEF recovery regardless of AIC status.

### Solving the ‘chicken‐and‐egg’ paradox: impact of atrial fibrillation burden

Our results provide substantial insights into the classic ‘chicken‐and‐egg’ paradox in patients who have concomitant persistent AF and LVSD. In particular, our results indicate that LVSD in this patient population is more likely to be secondary to, and induced by, the tachyarrhythmic condition of AF. The rates of AIC in patients with AF and LVSD have been reported in previous studies to be similar, ranging from 50% to 82%.^19^, ^20^, ^21^ The notion that AF plays a pivotal role in the development of HF in these patients is further supported by the higher prevalence of AIC.

These findings corroborate overwhelming evidence supporting the role of AF ablation in patients with AF and HF, which has recently received a Class I indication.^1^ Data from landmark trials including the CASTLE‐AF,^2^ CASTLE‐HTx,^3^ and CABANA^4^ trials unanimously show strong mortality benefits with AF ablation in this patient population.

The physiological sequelae of AF ablation include reduction in AF burden and thus overall HR reduction, improvement in HR variability, regression from persistent to paroxysmal AF, and decreased reliance on AADs.^22^ These effects combined likely contribute to reduced cellular calcium overload and neurohormonal activation, thereby promoting reverse remodelling of the ventricles.^23^, ^24^

Soulat‐Dufour *et al*.^25^ demonstrated that the maintenance of sinus rhythm in patients with AF and HF results in improved cardiac haemodynamics. This includes a reduction in all atrial indexed volumes, an increase in end‐diastolic left ventricular volume index, and an increase in end‐systolic right ventricular volume index, as well as improved ventricular function. In addition, the authors observed improved valvular regurgitation related to sinus rhythm, further highlighting the positive effects of strict rhythm control.^25^

In our study, we observed that the reduction of AF burden in all patients with persistent AF and LVSD, regardless of whether they fulfill the criteria for AIC or not and regardless of binary AF recurrence, is significantly correlated with LVEF improvement after ablation. On the other hand, HR reduction was not associated with AIC status. We hypothesize that this may be due to insufficient power or an intrinsic rhythm‐dependent rather than rate‐dependent mechanism in those patients. The latter hypothesis is supported by findings from the EAST‐AFNET 4 trial that showed superior outcomes with rhythm control compared with rate control, despite adequate rate suppression in both arms.^5^ Previous work by our group also showed that lower AF burden was associated with more reverse remodelling of the eft atrium, which in turn was associated with improved structural, functional, and electrical remodelling of the left atrium in general, and particularly in patients with persistent AF and LVSD.^26^ This reverse remodelling likely contributes to the observed improvements in cardiac function and underscores the therapeutic potential of AF ablation in this patient population.

### Role of atrial septal fibrosis

In the present study, we found that atrial septal regional fibrosis, rather than the total LA fibrosis quantity, is a significant predictor of AIC. Specifically, patients with non‐AIC had more fibrosis in the LA septum compared to the rest of the left atrium, while patients with AIC had less LA septal fibrosis compared to the rest of the LA wall.

The LA septum is a complex structure with an important role in the interatrial conduction of electrical activity. While the true interatrial septum's role in conduction is contested,^27^ nearby structures such as Bachmann's bundle and the coronary sinus contribute unique electrophysiological properties that facilitate interatrial conduction.^28^ We hypothesize that less atrial fibrosis near these conductive areas may enhance conduction of fibrillatory waves between the atria in AIC patients.^29^, ^30^ This worsens the mechanical function of biatrial contraction, amplifying the effects of atrial arrhythmias on ventricular function.^31^

Furthermore, enhanced interatrial conduction likely increases the irregularity of atrial input to the atrioventricular node. This would likely lead to increased ventricular rates, further deteriorating left ventricular function in AIC patients.^31^ AF ablation and the observed reduction of AF burden likely ameliorates these effects and leads to reverse remodelling of the left atrium and ventricle, as described above.

While the quantitative amount of atrial fibrosis on LGE‐CMR has previously been established as an important prognosticator for AF patients,^14^ recent work by our group emphasizes the critical role of regional distribution of atrial fibrosis.^18^ Conversely, ventricular fibrosis detected by LGE‐CMR was not predictive for AIC in multiple studies.^10^, ^32^ However, the CAMERA‐MRI (Catheter Ablation Versus Medical Rate Control in Atrial Fibrillation and Systolic Dysfunction) trial demonstrated that patients without ventricular LGE experienced more significant LVEF improvement post‐ablation.^21^ These conflicting results suggest a less critical role for ventricular fibrosis, while our findings emphasize the role of atrial fibrosis and its distribution as a driver of LVSD in patients with AIC, warranting further investigation.

### Challenges of current arrhythmia‐induced cardiomyopathy diagnosis

The current retrospective approach to diagnosing AIC is inadequate. This method, which diagnoses AIC only after observing the recovery of left ventricular systolic function following AF ablation, does not allow clinicians to fully understand the aetiology of HF before treatment. This lack of pre‐treatment diagnostic clarity can lead to suboptimal patient management and increased healthcare costs, as it prevents targeted interventions and prolongs uncertainty in treatment effectiveness.^8^ Several studies have proposed various and sometimes conflicting, methods to predict AIC. This inconsistency further complicates the ability to identify AIC accurately before treatment, highlighting the need for more reliable and prospective diagnostic tools.

### Other predictive factors for arrhythmia‐induced cardiomyopathy

A study by Nia *et al*.^33^ showed that a reduction in B‐type natriuretic peptide (BNP) levels 4 weeks after cardioversion significantly differentiated between AIC and non‐AIC in patients with supraventricular tachycardia. Their findings showed that a reduction in BNP levels was directly associated with improved ejection fraction, which, although accurate, does not offer the capability to assess the aetiology of HF before treatment. In contrast, our data did not show any statistical significance for pre‐ablation BNP levels in predicting AIC status.

Similarly, another study by Brembilla‐Perrot *et al*.^19^ indicated that AIC patients were generally younger, less likely to have ischaemic cardiomyopathy, and less likely to have a history of AAD therapy. In our study, age, history of coronary artery disease, and prior use of AADs were not significant predictors of AIC, while hyperlipidaemia did not reach statistical significance. It is important to note that Brembilla‐Perrot *et al*. studied 184 patients undergoing CA for atrial flutter, whereas our study focused on patients with persistent AF, which may explain the differences in these results.

### Limitations

The retrospective nature of this study has its intrinsic limitations to generalizability of our results. LVEF in the study was evaluated by either echocardiography or by cine MRI of the left ventricle, which may have introduced bias to the data. However, the percentage of patients assessed by echocardiography compared to MRI is similar between the AIC and non‐AIC patients, and any potential differences are equally distributed between the two groups. Furthermore, the DECAAF II imaging protocol, previously published and validated,^9^, ^14^, ^15^, ^16^ did not require the collection of ventricular MRI data or voltage map data. Comprehensive analysis of differences in left ventricular function between the different groups would provide substantial insights to this paper. The DECAAF II protocol did not require collection of medication doses, so we cannot ascertain whether patients were up‐titrated prior to, or after ablation. Medication data, including GDMT use, was collected retrospectively and may be incomplete due to variability in electronic medical record systems across international sites, potentially leading to underestimation of GDMT utilization. However, we expect any effect this may have to be equally distributed between the two groups.

## Conclusion

Our study highlights AF as an initiator and primary aetiology of LVSD in the majority of patients with concomitant persistent AF and LVSD. Furthermore, we defined atrial septal fibrosis as a pre‐ablation predictor of AIC status. Additionally, our findings reveal that LVEF improvement post‐ablation is closely associated with reduced AF burden, highlighting the interplay between rhythm control and cardiac systolic function. These insights not only enhance our understanding of the AF–HF relationship but also pave the way for more targeted and effective therapeutic strategies.

**Conflict of interest**: P.S. is member of the Advisory Board for Abbott, Biosense Webster, Boston Scientific and Medtronic. N.M. reports consulting fees from Biosense Webster, Boston Scientific and AtriCure; speaker honoraria from Abbott, Biosense Webster, AtriCure and Sanofi; research support from Abbott, Medtronic, Biosense Webster, Siemens, GE, Boston Scientific, Sanofi and Samsung; has a family member as the CEO of Cardiac Designs; is the founder of Marrek, is named in a patent issued for MRI fibrosis imaging, and is a previous shareholder of Cardiac Designs. C.S. received research support and lecture fees from Medtronic, Abbott, Boston Scientific, and Biosense Webster; is a consultant for Medtronic, Boston Scientific, and Biosense Webster; has received grant support from the Else Kröner‐Fresenius‐Stiftung and Deutsche Herzstiftung. All other authors have nothing to disclose.

## Supporting information


**Appendix S1.** Supporting Information.
