# Supplementary material for: Characterization of arrhythmia‐induced cardiomyopathy using magnetic resonance imaging in patients with persistent atrial fibrillation and left ventricular systolic dysfunction – insights from DECAAF II
Source: Eur J Heart Fail. 2025 May 12;27(9):1622–32. doi: 10.1002/ejhf.3684 (PMC12502454; doi:10.1002/ejhf.3684)
Supplement: Supplementary file 1 — Appendix S1. Supporting Information. [file EJHF-27-1622-s001.docx]

|  | Total | AIC | Non-AIC | P-value |
| --- | --- | --- | --- | --- |
| Number of GDMT Medications | 0.56 (0.88) | 0.60 (0.90) | 0.51 (0.86) | 0.5393 |
| Angiotensin converting enzyme inhibitors (ACEi), Angiotensin receptor blockers (ARB) or angiotensin receptor -neprilysin inhibitors (ARNI) | 27 (22.7%) | 19 (26.4%) | 8 (17.0%) | 0.2330 |
| Beta Blockers | 34 (28.6%) | 21 (29.2%) | 13 (27.7%) | 0.8588 |
| Mineralocorticoid Receptor Antagonists (MRAs) | 6 (5.0%) | 3 (4.2%) | 3 (6.4%) | 0.5891 |
| No Data on Sodium Glucose Cotransporter 2 Inhibitors (SGLT2i) was collected. SGLT2i were introduced as GDMT in the 2022 AHA/ACC/HFSA Guidelines for the Management of Heart Failure, and the DECAAF II trial was published in 2022 | | | | |

Supplementary Table 1: Number of patients on guideline-directed medical treatment (GDMT) for heart failure before ablation

|  | Total | AIC | Non-AIC | P-value |
| --- | --- | --- | --- | --- |
| Number of GDMT Medications | 0.79 (0.81) | 0.79 (0.95) | 0.79 (0.89) | 0.7795 |
| Angiotensin converting enzyme inhibitors (ACEi) or Angiotensin receptor blockers (ARB) or angiotensin receptor -neprilysin inhibitors (ARNI) | 42 (35.3%) | 29 (40.3%) | 13 (27.7%) | 0.1591 |
| Beta Blockers (carvedilol, bisoprolol, or metoprolol succinate) | 43 (36.1%) | 23 (31.9%) | 20 (42.6%) | 0.2389 |
| Mineralocorticoid Receptor Antagonists (MRAs) | 9 (7.6%) | 5 (6.9%) | 4 (8.5%) | 0.7521 |
| No Data on Sodium Glucose Cotransporter 2 Inhibitors (SGLT2i) was collected. SGLT2i were introduced as GDMT in the 2022 AHA/ACC/HFSA Guidelines for the Management of Heart Failure, and the DECAAF II trial was published in 2022 | | | | |

Supplementary Table 2: Number of patients on guideline-directed medical treatment (GDMT) for heart failure at the time of post-ablation left ventricular ejection (LVEF) measurement.

|  | Non-AIC | AIC | Total | P-value |
| --- | --- | --- | --- | --- |
| Class I: Sodium Channel Blockers | 1 (2.1%) | 2 (2.8%) | 3 (2.5%) | 0.8250 |
| Class II: Beta Blockers | 16 (34.0%) | 22 (30.6%) | 38 (31.9%) | 0.6900 |
| Class III: Potassium Channel Blockers | 3 (6.4%) | 13 (18.1%) | 16 (13.4%) | 0.0681 |
| Class IV: Non-dihydropiridine calcium channel blockers | 4 (8.5%) | 6 (8.3%) | 10 (8.4%) | 0.9728 |
| Class V: Other anti-arrhythmic drugs | 2 (4.3%) | 2 (2.8%) | 4 (3.4%) | 0.6620 |

Supplementary Table 3: Number of patients on anti-arrhythmic drugs before ablation

|  | Non-AIC | AIC | Total | P-value |
| --- | --- | --- | --- | --- |
| Class I: Sodium Channel Blockers | 3 (6.4%) | 2 (2.8%) | 5 (4.2%) | 0.3379 |
| Class II: Beta Blockers | 22 (46.8%) | 23 (31.9%) | 45 (37.8%) | 0.1021 |
| Class III: Potassium Channel Blockers | 10 (21.3%) | 19 (26.4%) | 29 (24.4%) | 0.5254 |
| Class IV: Non-dihydropiridine calcium channel blockers | 10 (21.3%) | 8 (11.1%) | 18 (15.1%) | 0.1303 |
| Class V: Other anti-arrhythmic drugs | 1 (2.1%) | 2 (2.8%) | 3 (2.5%) | 0.8250 |

Supplementary Table 4: Number of patients on anti-arrhythmic drugs at the time of post-ablation left ventricular ejection (LVEF) measurement.
